# Variation Range of Different Inductor Topologies with Shields for RF and Inductive Sensing Applications

**DOI:** 10.3390/s22093514

**Published:** 2022-05-05

**Authors:** Fares Tounsi, Mohamed Hadj Said, Margo Hauwaert, Sinda Kaziz, Laurent A. Francis, Jean-Pierre Raskin, Denis Flandre

**Affiliations:** 1 Sensors, Microsystems and Actuators Laboratory of Louvain (SMALL), Université Catholique de Louvain, Place du Levant 3, 1348 Louvain-la-Neuve, Belgium; margo.hauwaert@uclouvain.be (M.H.); laurent.francis@uclouvain.be (L.A.F.); jean-pierre.raskin@uclouvain.be (J.-P.R.); denis.flandre@uclouvain.be (D.F.); 2 Systems Integration & Emerging Energies (SI2E), National Engineering School of Sfax, University of Sfax, Route Soukra, BP 1173, Sfax 3038, Tunisia; mohamed.hadjsaid@crmn.rnrt.tn (M.H.S.); sinda.kaziz@fsm.u-monastir.tn (S.K.); 3 Center for Research in Microelectronics and Nanotechnology (CRMN), Sousse 4050, Tunisia

**Keywords:** planar inductor topologies, eddy-current microsensor, tuned inductors, proximity measurement, ferromagnetic resonance (FMR), non-destructive evaluation

## Abstract

In this study, different planar inductor topologies were studied to evaluate their characteristic parameters’ variation range upon approaching Fe- and Cu-based shield plates. The use of such materials can differently alter the electrical properties of planar inductors such as the inductance, resonant frequency, resistance, and quality factor, which could be useful in multiple devices, particularly in inductive sensing and radio-frequency (or RF) applications. To reach an optimal design, five different square topologies, including spiral, tapered, non-spiral, meander, and fractal, were built on a printed circuit board (PCB) and assessed experimentally. At the working frequency of 1 MHz, the results showed a decrease in the inductance value when approaching a Cu-based plate and an increase with Fe-based plates. The higher variation range was noticeable for double-layer topologies, which was about 60% with the Cu-based plate. Beyond an intrinsic deflection frequency, the inductance value began to decrease when approaching the ferromagnetic plate because of the ferromagnetic resonance (FMR). It has been shown that the FMR frequency depends on the inductor topology and is larger for the double-layer spiral one. The *Q*-factor was decreasing for all topologies but was much faster when using ferromagnetic plates because of the FMR, which intensely increases the track resistance. The resonant frequency was increasing for all double-layer topologies and decreasing for single-layer ones, which was mainly due to the percentage change in the stray capacitance compared to the inductance variation. The concept of varying inductors by metal shielding plates has great potential in a wide range of nondestructive sensing and RF applications.

## 1. Introduction

Inductors are elementary but important electrical elements in many radiofrequency (RF) [1], wireless communication/power transfer [2], and magnetic sensing systems [3]. In integrated RF circuits, planar inductors are an attractive solution for the achievement of transceiver/receiver modules, power amplifiers, power splitters/dividers and combiners, voltage-controlled oscillators (VCOs), and filters [4]. Though, some inductor characteristics could be varied, or tuned, which adds extra functionalities and features in many electronic circuits and systems. For instance, tuned inductors are very advantageous for self-adjusting matching networks in RF circuits, where the tuning mechanism is often implemented as part of an LC circuit and where any change in the inductance value leads to altering its resonance frequency [5]. Many methods could be used to vary the inductance value and alter inherent properties in both micro- and macro-scale technologies; some manipulate the magnetic flux or mutual inductance; many modulate the core properties; and a significant number of works involve shortening the inductor length. Tunable inductance has the advantage of the quality factor and bandwidth control, low voltage operation, and a potentially larger tuning range [6]. More specifically, varying or tuning the inductor can be achieved through four means: (i) inductive coupling variation based on a change in the mutual inductance coupling coefficient between the primary and secondary windings of a split inductor via bimorph actuators [7,8]; (ii) magnetic core tuning based on changes in core properties such as permeability, (by magnetoelectric effect (ME) [9,10], magnetoelastic coupling [11], or magneto-impedance effect [12]), DC current bias, position [13], etc.; (iii) a discretely-tuned length, which could be realized using micro relays [14,15], micro-switches (MOSFET) [16], or liquid (e.g., mercury) moving inter-spires [17] to vary the effective path length of the current through the structure, leading to a reduction in the stored magnetic energy, and thus the inductance; and (iv) the metal shielding tuned (MST) approach, realized using a movable metal plate so that inductance variation results from changes in the inductor magnetic flux [18,19,20].

For RF integrated-circuit applications, inductor tuning must be accurate (error <2%) with high quality factors (>15) and self-resonance frequencies (>10 GHz), and it must operate with low losses, low power consumption, and high linearity [21]. The requirements are much less severe in proximity magnetic sensing applications founded on the MST approach, where planar variable inductors are used in many nondestructive evaluation (NDE) applications [22,23,24,25]. These sensors exploit the electromagnetic induction principle to detect fluctuations in a magnetic field initially generated by a reference inductor, for non-contact measurement of displacement, distance, position, oscillations, and vibrations. In fact, the opposite magnetic field due to the eddy current creation can be measured, and its amplitude and duration can provide extremely precise measurements regarding the proximity or thickness of any nearby conductive material. Printed Circuit Board (PCB)-based inductors are also reported for NDE magnetic sensors to evaluate the material propriety in near-surface configurations such as meander and mesh types [26]. PCB-based inductors are gaining appeal in proximity sensing applications due to their various advantages over conventional 3D inductors, such as their small size, low cost, batch fabrication, manufacturability on flexible substrates, simplicity, and durability [27,28]. However, most works have focused on studying single-layer inductor geometries without paying attention to either other emerging topologies or on-chip fabrication rules [29,30], which could be required in some highly integrated specific applications. Therewith, no comparison has been made so far on the consequences of different types of metal plates on the variation of all inductance properties; although, diverse shielding types were used in the bibliography to achieve inductance tuning [19,31]. Consequently, we will examine different inductor topologies patterned on PCBs with the aim of comparing their variation ranges and behavior upon shielding.

The article is organized as follows: in the second section, the five PCB-based inductor topologies are presented and numerically studied to evaluate their lumped electrical parameters. All topologies were evaluated using the Ansys HFSS with a finite element method (FEM) solver. Next, tuning physics theory and the equivalent circuit of the MST approach are detailed. In the fourth section, three plates with different relative permeabilities are used as shielding to vary the inductance values. Manufactured elements were characterized using a vector network analyzer. The last section presents the measured performance variation in order to explore the most sensitive topology that could be used in RF and proximity sensing applications.

## 2. Topologies Presentation and Simulation

**Figure 1:**
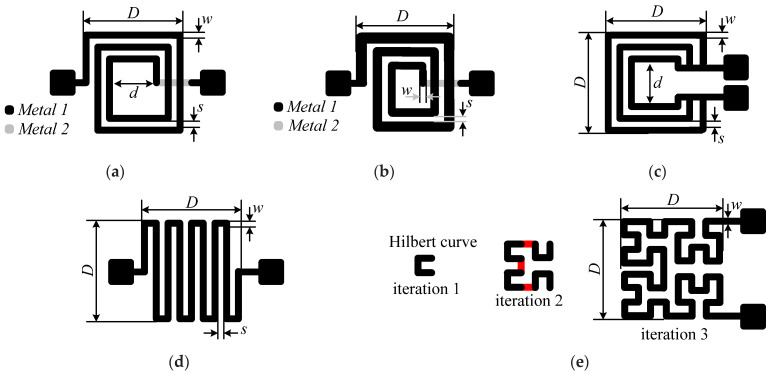
Evaluated square planar topologies: (**a**) spiral, (**b**) tapered, (**c**) non-spiral, (**d**) meander, and (**e**) fractal (based on Hilbert curve: iteration 1, iteration 2–connections are in red–, and iteration 3).

Planar inductors could be classified into single-layer and double-layer (or multilayer, in a general context) types. The technological realization of the double-layer inductors needs two levels of metallization (spiral and underpass) as well as two vias [32]. The double-layer spiral inductor (Figure 1a) is the most popular in RF applications due to the high achievable inductance values per unit of wire length. This feature is achieved thanks to its rolled layout design, which approaches ordinary ones, promoting positive mutual inductance contributions between neighboring conductors in the resulting inductance value. Spiral structures with multiple turns tend to have higher resistive losses in the inner turns. The reason behind this is that the magnetic flux increases as we approach the center of the spiral because of the addictive nature of the flux of each successive loop of the spiral. For this reason, hollow and tapered spirals are advantageous techniques [33]. Hence, once the width of the structure is tapered, as shown in Figure 1b, the performance of the spiral can be improved [34]. The latter represents a topology where the width decreases when going towards the interior of the spiral. Since the wide inner turns do not lower the resistance (due to current constriction), it is best to transfer the width to the outer turns while keeping the footprint of the spiral constant.

Single-layer planar inductor topologies, such as meander, non-spiral, and fractal, are also examined in this work. Single-layer types have both contact pads and an inductor layout on the same level, as shown in Figure 1c–e; thus, only one metal layer during fabrication is required [32]. Hence, there is no need for an internal metal via contact as in two-layer spiral inductors. In meander inductors, as shown in Figure 1c, the current vectors of adjacent tracks are opposite, causing a negative mutual inductance. Therefore, its inductance value is about three to eight times lower (depending on dimensions) compared to the value achieved with a spiral geometry of the same effective track length. Non-spiral inductors, shown in Figure 1d, are advantageous in terms of energy consumption as their resistance is lower than the spiral ones. The low resistance is achieved due to the short-circuited turns’ configuration, which causes a non-homogeneous distribution in the magnetic field. This is due to the asymmetrical current division between the turns of the inductor since they are interconnected as a set of short-circuited parallel conductors. Hence, the innermost turn has the least resistance, so the majority of the input current flows through it, which makes the magnetic field maximal in the vicinity. So, the resistance of the turns increases gradually towards the outer turns, which in turn leads to a decrease in the magnetic field towards the outer turns. Fractal inductors are a whole new set of geometrical objects featuring two main common properties, which are self-similarity and the fractional dimension. Fractal-based inductor design is a method of achieving high inductance values in the minimum footprint area when using a single-layer topology. The key aspect of higher-level fractal inductors lies in the repetition of a motif over two or more scale sizes, referred to as iterations. Based on Hilbert’s space-filling curve in a two-dimensional plane, fractal inductors are capable of achieving an increased value of inductance without increasing their footprint. A third-order Hilbert fractal inductor, based on three iterations of the Hilbert curve, is represented in Figure 1e. The fractal design is competitive at lower iterations, but as the self-similarity increases, designs yield lower values of the quality factor. Low-cost fractal inductors represent a potential design for flexible/stretchable electronics thanks to their short tracks, where highly deformable inductors have applications in medical monitoring and strain sensing [35]. Next, a numerical extraction of the inductors’ parameters will be carried out with the Ansys HFSS^®^ (an acronym for High Frequency Simulation Software) on the basis of a finite element analysis (FEA).

Initially, the five aforementioned inductor topologies are simulated, and their electrical characteristics are extracted, i.e., the equivalent lumped-element model, the resonant frequency, and the quality factor. Figure 1 illustrates the five topologies, and Table 1 lists their geometric dimensions, where *n* is the turn’s number; *D* and *d* are the outer and inner diameters, respectively; and *w* and *s* are the track width and spacing, respectively. The inductors’ dimensions have been chosen as small as possible for drilling on the PCB. The tapered inductor track increased by 50 µm with each turn. All fabricated structures have the same footprint of *D × D*. These topologies were selected because they are among the most common in the design of inductive sensors and RF circuits, combined with relative ease of manufacture.

**Figure 2:**
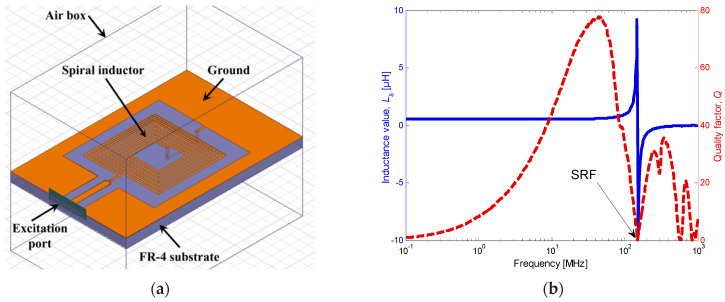
Spiral inductor topology: (**a**) design in HFSS and (**b**) simulated inductance variation and quality factor versus frequency.

Up to the resonant frequency, all inductor topologies can be modeled by an inductor *L_s_* in series with a resistor *R_s_*, both in parallel with a parasitic capacitor *C_s_*. The numerical modeling was carried out using Ansys HFSS to extract each inductor’s parameters. Figure 2a shows the layout of the spiral inductor placed on an FR-4 substrate and surrounded by an air box. The air box dimensions should be 10 times greater than the studied component dimensions to consider all radiated fields. The boundary conditions are defined at the edge of the box by a radiation limit that allows the field to propagate infinitely far into space. The inductance, resistance, and quality factor values can be found from the admittance matrix (or *Y*-matrix) when using *Y*_11_ obtained from the simulation as given by:

(1) $$L_{s}=\frac{ℑ\left[1/Y_{11}\right]}{\omega }$$

(2) $$R_{s}=ℜ\left[{\left(Y_{11}\right)}^{-1}\right]$$

(3) $$Q\left(\omega \right)=\frac{ℑ\left(-Y_{11}\right)}{ℜ\left(Y_{11}\right)}$$

where *Y*_11_ is extracted, or measured with a vector network analyzer, from a simple conversion of the simulated scattering matrix *S*_11_ as:

(4) $$Y_{11}=\frac{\left(1-S_{11}\right)}{\left(1+S_{11}\right)}Y_{0}$$

where *Y*_0_ is the characteristic admittance. The variation in the inductance and the quality factor of the spiral inductor, extracted from the S-matrix, are shown in Figure 2b. The self-resonant frequency (*SRF*), simulated at about 160 MHz, depends on the inter-winding stray capacitance, *C_s_*, and the inductance value, *L_s_*, as:

(5) $$SRF=\frac{1}{2\pi \sqrt{L_{s}C_{s}}}$$

From Figure 2b, it can be noticed that the simulated quality factor *Q* increases up to 78 at 41 MHz and then drops to zero at the *SRF*. At resonance, the value of the inductance will rise, and, beyond that, the circuit will switch to capacitive behavior.

## 3. Tuning Physics and Equivalent Lumped Circuit

**Figure 3:**
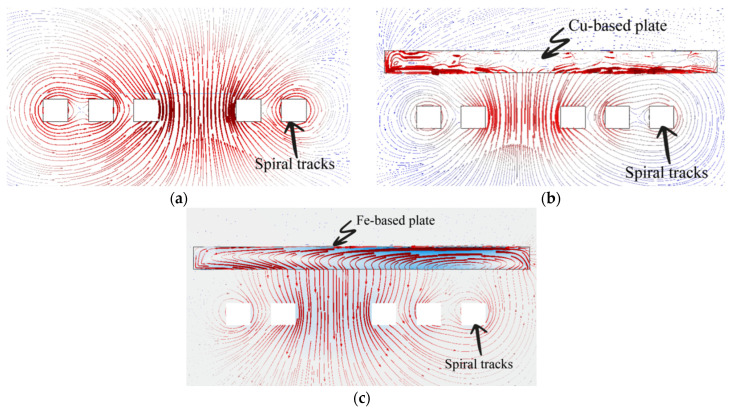
Module and direction distribution of the tangential magnetic field (thicker lines reflect higher intensity): (**a**) without shielding, (**b**) with Cu-based shielding, and (**c**) with Fe-based shielding.

The tuning approach applied to different planar inductors is based on the metal shielding tuning (MST) method, which consists of approaching conductive plates/ribbons of 25 × 25 mm^2^-size and 17 µm-thick. In experiments, the inductance variation is assessed when using two different types of plates based on iron (Fe) and copper (Cu). Nanocrystalline (*µ_r_* = 80,000, *ρ* = 115 µΩ cm, with a saturation flux density of 1.25 T) and amorphous (*µ_r_* = 5000, *ρ* = 130 µΩ cm, with a saturation flux density of 1.56 T) soft ferromagnetic Fe-based ribbons are compared to a Cu-based plate with a relative permeability slightly lower than that of the air (i.e., *µ_r_* = 0.999994) and *ρ*_Cu_ = 1.68 µΩ cm. Permeability reflects the material’s ability to pass magnetic lines of flux or even a high magnetic susceptibility. In contrast to permanent magnets, the remanent magnetization of a ferromagnetic material is very small when no external field is applied. Without shielding, the distribution of the tangential magnetic field module (contour map) and direction (arrow) are shown in Figure 3a, where the maximum field strength is around the center. In Figure 3b,c, a shielding metal plate above a planar spiral inductor is applied. In this setup, two physical approaches are at stake. When a Cu-based plate approaches the magnetic field induced by the planar inductor, some of the magnetic energy is transferred into the conductive plate (Figure 3b). This latter causes a circulating electrical current known as an eddy current. Based on Lenz’s law, a counteractive magnetic field will be induced in the vicinity of the inductor, which results in a reduction in the effective inductance due to the opposing induced magnetic flux, which will add up to the initial flux due to the inductor actuation. In contrast, at low frequencies, as the Fe-based ferromagnetic plate gets closer to the inductor, it will bring about a change in the magnetic flux lines that will penetrate the ferromagnetic plate. As a result, the ferromagnetic shield will increase the original magnetic field, which in turn will lead to an increase in the magnetic energy stored in the inductor, and consequently the inductance value will be increased [36]. The principle of the variable inductor is depicted in Figure 3c, where the magnetic field lines close upon approaching a ferromagnetic plate.

**Figure 4:**
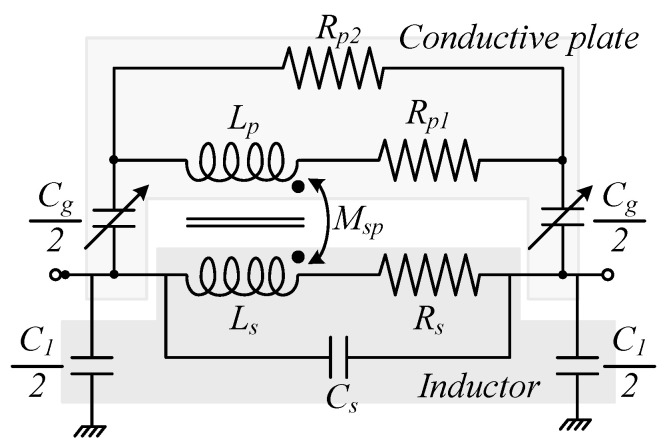
Lumped physical model of the variable planar inductor implemented on a FR-4 substrate with a proximity plate.

The two-port equivalent circuit model of the variable inductor structure is depicted in Figure 4. The latter includes an RLC lumped equivalent circuitry (*L_s_*, *R_s_*, and *C_s_*) representing the PCB inductor, in addition to the shielding plate electrical model composed of an inductor in series with a resistance (*L_p_*, *R_p_*_1_), both in parallel to a conductive part (*R_p_*_2_). The two-port lumped equivalent model takes into consideration the parasitic capacitance between the spiral inductor and the shielding metal plate (*C_g_*), in addition to the track to ground plane capacitances (*C*_1_). Finally, *k_sp_* is the equivalent coupling coefficient between the movable shielding metal plate and the spiral inductor defined by:

(6) $$k_{sp}=\frac{M_{sp}}{\sqrt{L_{s}L_{p}}}$$

where *M_sp_* is the equivalent mutual inductance between the inductor and the shielding plate. The coupling coefficients *k_sp_*, *M_sp_*, and *C_g_* are functions of the gap *g*, while the other parameters are not [31].

## 4. Inductors Fabrication and Experimental Results

**Figure 5:**
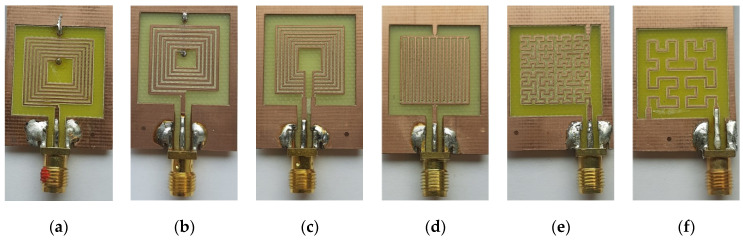
Different inductor topologies patterned on PCB interfaced via SMA connector: (**a**) spiral, (**b**) tapered, (**c**) non-spiral, (**d**) meander, (**e**) fourth-order fractal, and (**f**) third-order fractal.

All planar inductors were patterned on an FR-4-type board (17 µm copper thickness and 1.7 × 10^−8^ Ω·m as resistivity) with a minimal drill bit resolution equal to 400 µm. In addition to the four basic topologies, two kinds of fractal inductance were analyzed, which are those of fourth- and third-order (Figure 5). The inductors were characterized using a Vector Network Analyzer ENA E5080A from Keysight connected via a 50 Ω-input impedance 3.5 mm SMA connector. The ENA collected the scattering matrix S data within frequencies ranging between 100 kHz and 1 GHz, and then the lumped-element model parameters were extracted using real and imaginary parts of the admittance *Y*_11_ [32]. Table 2 identifies different electrical parameters of all inductor topologies, extracted from measurements and simulations. As we can see, the measured values show quite sufficient agreement with the simulated ones. The difference is due to a ±5% inaccuracy in the resulting track width. It is worth noting that the inductance value of the spiral inductor is the highest, which is due to the high magnetic field flux generated between its different spires. However, the latter clearly exhibits a high value of parasitic parameters (*R_s_* and *C_s_*), which have a significant impact on the quality factor and the resonant frequency compared to the non-spiral one [37]. Despite its very low inductance, the non-spiral inductor has the best quality factor compared to other planar inductors. In fact, due to its very low resistivity, the non-spiral inductor is showing interesting properties in terms of SFR, the quality factor, and loss parameters, which is advantageous in some multiband RF applications. Lastly, the meander inductor represents the higher losses in terms of resistance and capacitance, and hence the *Q*-factor is the lowest. The next paragraph will be devoted to a comparison of the yields of different topologies in terms of inductance and *Q*-factor variation when using different plate types.

**Figure 6:**
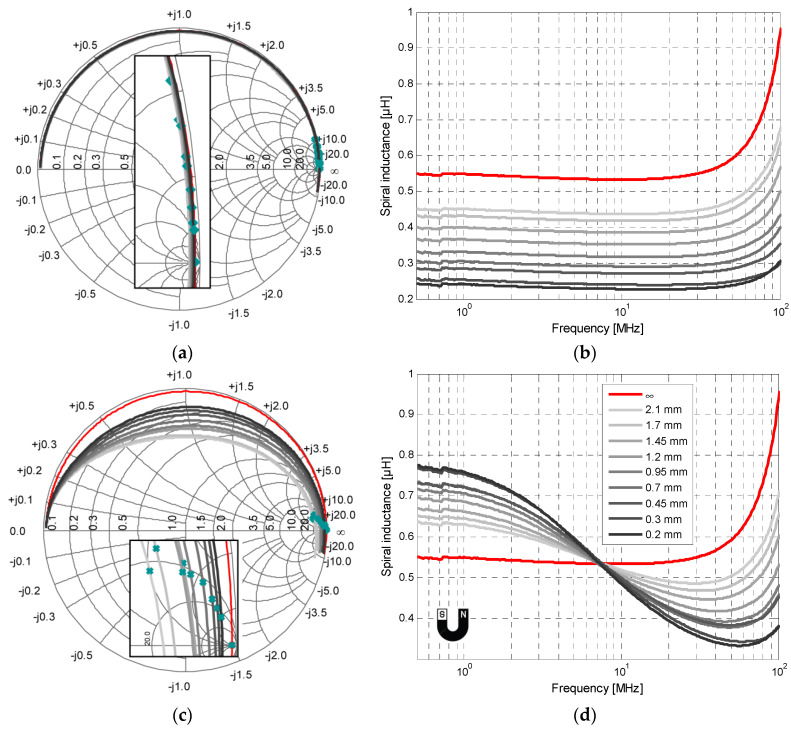
Spiral inductor measurement: with Cu-plate (**a**) *S*_11_ parameter drawn in Smith chart, and (**b**) inductance variation versus frequency, with Fe-plate (Nanocrystalline); (**c**) *S*_11_ parameter drawn in Smith chart; and (**d**) inductance variation versus frequency (the legend on (**d**) applies for all subgraphs).

The setup approach consists of intercalating, for each measurement, a 3D drilled sheet with the appropriate thickness between the inductor and the floating-bias shielding plates [38]. The sheet is a 3D polymer printed support used to accurately control the separation gap. Eventually, air with a loss tangent of ~0.06 is sandwiched between the planar inductor and the shielding. Figure 6a,b present the *S*_11_ parameter and the inductance value over frequency when varying the gap between the Cu-based metal plate and the spiral inductor, respectively. The separation distance ranged from 0.2 mm to 2.1 mm. The inductance was extracted from the S-parameter when using Equation (1). As seen in Figure 6b, the inductance value decreased over the entire frequency range as the gap *g* decreased. As explained beforehand, when approaching the Cu-based plate, a magnetic field in the opposite direction appeared due to the eddy current. Based on Lenz’s law, the inductor’s stored magnetic energy will be reduced as well, and hence the inductance will be decreased. In the course of the Cu-plate approaching, real and imaginary parts of the inductor impedance are drawn in Figure 6a for gaps from 0.2 mm up to 2.1 mm. As shown in the Smith chart, the *S*_11_ real part remains almost constant over frequency for all separation distances.

In contrast to the copper plate case, at low frequencies, when approaching the ferromagnetic plate, the magnetic field is confined to the vicinity of the inductance. This in turn increases the inductor’s stored magnetic energy and hence the inductance value as expected. As the frequency increases, the real part of the inductor impedance increases immensely, as shown in the Smith chart in Figure 6c. Because of the high electrical conductivity of the high-µ plates, eddy currents tend to circulate in the shielding when the circuit is driven more in the RF range and generate a magnetic field that opposes the change in the one that created it, thus reacting back to the source according to Lenz’s law. In addition, the eddy currents generate resistive losses in the ferromagnetic shield and significantly lower its effective permeability, *µ_r_* [36]. As a result, the roll-off in inductance and the peaking in resistance with increasing frequency are due to the ferromagnetic resonance (FMR) in the high-µ shield film. This was originally described by Griffiths as the anomalous high-frequency resistance of ferromagnetic metals in which a large increase in the product of magnetic permeability and electrical resistivity was observed in certain magnetic fields depending on the excitation frequency [39]. Beyond the FMR frequency, the permeability of the ferromagnetic film drops below zero [36]. Because the FMR frequency in the high-µ plate depends on the B-field strength, there exists an intrinsic deflection frequency for each inductor topology at which the inductance value remains constant regardless of the separation distance between the plate and the inductor (Figure 6d). Prior to this deflection frequency, the inductance value would increase when bringing the high-µ plate closer, and beyond it, the opposite. Table 3 summarizes the measured deflection frequencies for different inductor topologies when approaching both ferromagnetic plates. This deflection frequency is all the greater as the permeability of the ferromagnetic plate is higher. Moreover, it could be highlighted that the double-layer inductors, whose induced magnetic flux is higher, possess a lower deflection frequency, which proves that the higher the magnetic flux, the more the effect of the ferromagnetic resonance occurs at a lower frequency. Based on the measured *S*_11_-parameter curve of the spiral inductor, in Figure 6c, it can be seen that the real part of the equivalent circuit drops crucially at the *SRF* as the gap is narrowing, which reflects an increase in ohmic loss. This will be translated into a significant loss of the quality factor, driven by the rise in the circuit’s resistance with frequency.

## 5. Results Extraction, Discussions, and Interpretations

**Figure 7:**
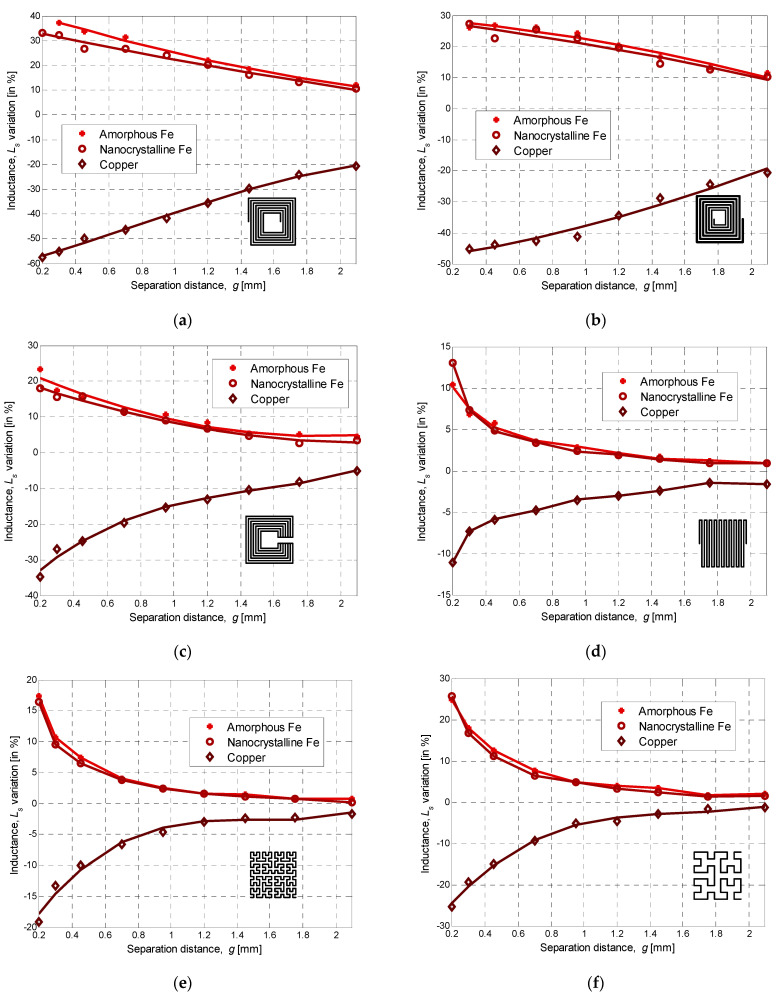
Measured inductance variation over separation distances for different planar topologies at 1 MHz: (**a**) spiral, (**b**) tapered, (**c**) non-spiral, (**d**) meander, (**e**) fourth-order fractal, and (**f**) third-order fractal.

Three main features characterize the performance of inductors: the inductance value, the quality factor (*Q*-factor), and the self-resonant frequency (SRF) [29]. Most often, the inductance, representing the sensor-sensitive core, is connected in parallel with an external capacitor, the combination of which is called a tank circuit. The change in the sensor’s inductance causes a shift in the resonant frequency of the LC circuit, which changes the amplitude of the output signal. The latter could be measured by a microcontroller to detect the presence of the metal target in the proximity-sensing distance. Hence, an increase in the sensor inductance due to a distancing/rapprochement will be translated into a shift in the resonant frequency of the tank circuit. Figure 7 shows the inductance variation taken at 1 MHz of the various manufactured inductance topologies for the same separation range. The step size was increased logarithmically between 100–350 µm, and measurements were taken twice for each inductor across the entire displacement range when using the three different types of plates. From Figure 7, it can be seen that approaching the Fe-based nanocrystalline or amorphous ribbons increases the inductance value, while the Cu-plate decreases it. In fact, when ferromagnetic plates are subjected to an external low-frequency magnetic field, a strong magnetization in the direction of the applied magnetic field is created, and the inductance B-field is then amplified, which results in a high inductance variation. Typically, ferromagnetic materials with higher magnetic permeability result in a greater positive susceptibility to an external magnetic field. However, the saturation flux density is critical and should be as high as possible because it gives the highest possible force line density. Accordingly, the inductance variation under the amorphous ribbon is slightly higher than under the nanocrystalline one since it has a higher saturation. The spiral topology has the highest variation range among double-layer designs, which is approximately 40% at 1 MHz and *g* = 0.2 mm, whereas the non-spiral inductance has the highest range for single-layer designs. On the other hand, we can see that the influence of the copper plate is greater at 1 MHz for all topologies than ferromagnetic ones, especially for the spiral square inductor, which changed by up to 60%. The tapered spiral inductor performs very closely to the spiral inductor. Single-layer inductors perform nearly half as well as double-layer inductors. The meander inductor presents the lowest variation range, barely approaching 15% for *g* = 0.2 mm.

As a conclusion, we can note that at 1 MHz, Fe-based ribbons induce a lower variation range compared to the copper plate, and the double-layer inductors are the most sensitive. In addition, their decay slope is almost linear compared to single-layer inductors, which could result in good sensor/circuit performance. This feature is also valid for non-spiral inductors where the measurement range is higher than the other single-layer topologies. It should be highlighted that these results are specific to the 1 MHz frequency, since at 100 kHz the range of variation increases when using the ferromagnetic plate and drops with the copper plate since the eddy current effect diminishes. At all frequencies, the spiral inductor, with its larger variation range, is the most responsive for displacement sensing applications, but it is also more complex in terms of manufacturing. However, design considerations depend very much on the application. For instance, for high resonant frequency applications, the non-spiral topology might be considered as the more suitable candidate.

**Figure 8:**
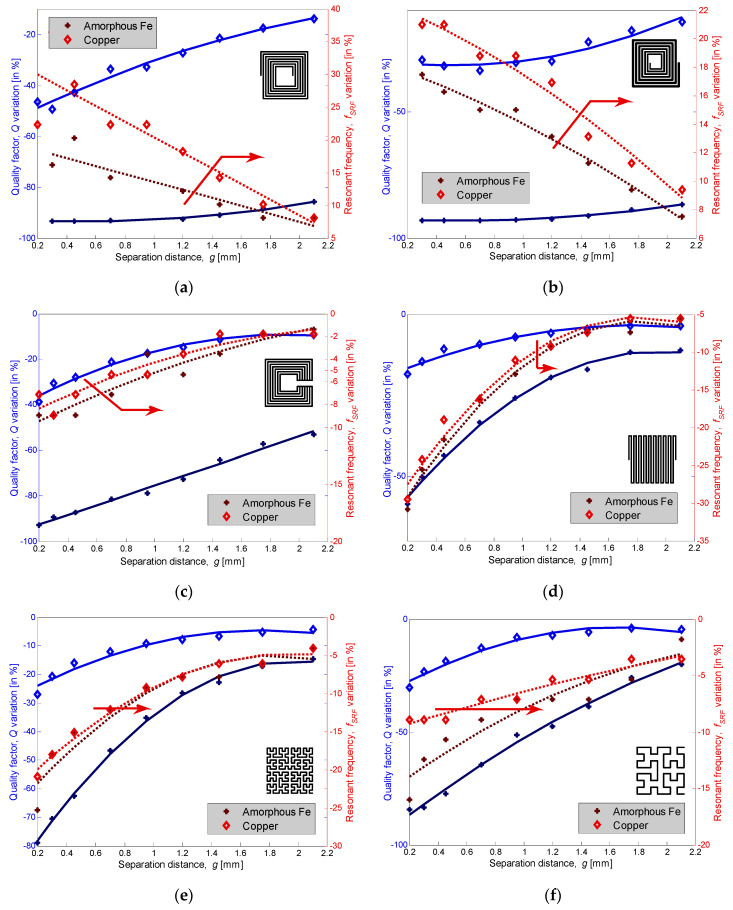
Variation range sensitivity percentage (quality factor in solid lines plotted on the left *y*-axis and resonant frequency in dashed lines plotted on the right one) versus different distances at 1 MHz: (**a**) spiral, (**b**) tapered spiral, (**c**) non-spiral, (**d**) meander, (**e**) fourth-order fractal, and (**f**) third-order fractal.

The *Q*-factor is the most important characteristic that determines the performance of inductors and, ultimately, the quality of devices within which they are implemented. The higher the *Q*-factor of the inductor, the closer it approaches the behavior of an ideal inductor. For instance, the primary concern in VCOs is to reduce phase noise as well as power consumption; both can be done by improving the *Q*-factor. For different assessed inductor topologies, the quality factor and self-resonant frequency variations are shown in Figure 8 for copper and amorphous plates. It can be seen that the *Q*-factor was decreasing when approaching either copper or iron plates. This was due to the increase in both the parasitic capacitance, *C_g_*, and the coupling coefficient, *k_sp_*, that appeared between the inductance and the metal plate, contributing to the degradation of the inductance quality. However, the *Q*-factor dropped faster at roughly double with ferromagnetic plates. The *Q*-factor is a quantitative measure of the inductance efficiency and is given as the ratio of its inductive reactance to its resistance at a given frequency (Equation (3)). In the case of a copper plate, the inductance decreased with the gap narrowing, while the resistance did not vary much since the skin effect is not that effective at the working frequencies. Therefore, the *Q*-factor variation range was less than 50% for all the topologies and was higher for the double-layer inductors. On the other hand, with ferromagnetic ribbons, the tremendous drop in the *Q*-factor was due to the FMR, which induces losses in the high-µ shield as its relative permeability drops to unity and the loss tangent reaches its peak value. Hence, the FMR sets the upper operating frequency instead of the self-LC resonance from the parasitic capacitance between the windings, magnetic shielding, and inductor layouts [10]. The worst degradation in the *Q*-factor was perceived for the spiral topology, since it has the lowest FMR and a larger variation range than the non-spiral one. In fact, the impedance’s real part increased considerably for double-layer topologies, which leads to a grave and steady diminution in the *Q*-factor even for large separation distances, as seen in Figure 8. Increasing the FMR frequency can be achieved by choosing a ferromagnetic material with a higher anisotropy field and/or higher saturation magnetization, using a laminated magnetic core, controlling the geometric structure to minimize eddy currents, and choosing a better inductor topology to have a good compromise between the FMR frequency and the desired inductor performance.

Regarding the SFR, which is the main factor that determines the operating frequency band, it can be seen from Figure 8 that for both plate-types, the shift is maximum when the gap is at its minimum. In addition, it should be emphasized that with the gap narrowing, the SFR is increasing for double-layer inductors and decreasing for single-layer ones. From Equation (5), the SFR is inversely proportional to the product of the equivalent inductance (which will decrease beyond the deflection frequency) and the circuit capacitance (which will increase with the rapprochement). Thus, the SFR’s variation will be determined by the term with the largest instability. In the case of single-layer inductors, the effect of increasing the equivalent capacitance is more dominant than the fact of decreasing *L*, which explains the reduction in the SFR. In this topology, the equivalent capacitance is formed only by the sum of the inter-track’s capacitance with *C_g_*. In double-layer inductors, the interlayer capacitance is added to the total capacitance, which reduces its volatility. In this case, the decrease in inductance is then more dominant than the increase in capacity, which explains the increase in the SFR. Consequently, the SFR rises for double-layer inductors and diminishes for single-layer inductors with the separation distance. Two zoomed windows in Figure 6a,c illustrate the increase in resonant frequency for the spiral inductor by showing the evolution of the same point (taken at the resonance frequency in the normal state–marked in green) when bringing the plate closer to the inductor.

## 6. Conclusions

The variation range of five PCB-made planar inductor topologies, including spiral, tapered, non-spiral, meander, and fractal, was investigated in this study. The electrical parameters of these topologies were confirmed using experimental measurements and FEM simulations. In the tuning evaluation setup, all inductors were approached by both Fe- and Cu-based metal shields over distances ranging from 0.2 mm to 2.1 mm. The Cu-based plate reduced the original magnetic field, therefore reducing the inductance value due to the counteractive magnetic field induced by the eddy current. This is in contrast to ferromagnetic plates, which confine the original magnetic field and then increase the inductance value at low frequencies. An intrinsic deflection frequency, related to ferromagnetic resonance (FMR), was found for each topology, from which the value of the inductance decreased when approaching the high-µ plates. The spiral inductor exhibited a large tuning range of up to 60% at 1 MHz with a Cu-based plate, together with an increase in the operating bandwidth. The *Q*-factor decreased for all topologies when approaching a shield, and especially for Fe-based plates, because of the FMR, which induces a dramatic increase in the real part component. In fact, with low electrical resistivity of ferromagnetic plates, eddy current loss will become a crucial problem. The resonant frequency was increasing for all double-layer topologies and decreasing for single-layer ones, which was mainly due to the percentage change in the stray capacitance compared to the inductance variation. The study highlights that the FMR frequency depends on the inductor topology and that it is larger for the double-layer spiral one.

## Figures and Tables

**Table 1:** Common dimensions of all fabricated planar inductor topologies.

Parameter	*n*	*D*	*d*	*w*	*s*
Value	6.5	15.6 mm	5.2 mm	0.4 mm	0.4 mm

**Table 2:** Measured/simulated initial electrical parameter values of different inductor topologies alone.

Parameter/ Topology	L_s_ (nH) (@1 MHz)		R_s_ (Ω) (@1 MHz)		*SRF* (MHz)		Quality Factor (*Q*)	
	Simul	Meas	Simul	Meas	Simul	Meas	Simul	Meas
Spiral	593	562	0.75	0.6	160	154	78 @41 MHz	63.27 @49.07 MHz
Tapered	461	428.2	0.5	0.32	181	175	104 @56 MHz	79 @59.3 MHz
Non-spiral	17.7	21.32	0.038	0.024	1000	1037	109 @91 MHz	100 @194 MHz
Meander	103.3	115.7	0.75	0.38	491	458	43 @151 MHz	22 @144.9 MHz
Fourth-order fractal	86.5	111.4	0.61	0.36	450	426	50 @120 MHz	30.76 @154.2 MHz
Third-order fractal	68	86.21	0.3	0.192	500	490	66 @115 MHZ	42.41 @163.4 MHz

**Table 3:** Deflection frequencies of different inductance topologies when approximated by ferromagnetic plates.

Inductor Topology		Spiral	Tapered	Non-Spiral	Meander	Fourth-Order Fractal	Third-Order Fractal
Deflection frequency (MHz)	Amorphous plate	7.17	7.06	13.4	14	22.35	19.17
	Nanocrystalline plate	7.41	7.31	13.6	20.25	27.62	19.83

## Data Availability

The data presented in this study are available on request from the corresponding author. Some results are discussed but not drawn due to page limitations.

## References

[1] Burghartz J.N. (2003). On the design of RF spiral inductors on silicon. IEEE Trans. Electron Devices.

[2] Batraa T., Schaltz E. (2015). Passive shielding effect on space profile of magnetic field emissions for wireless power transfer to vehicles. J. Appl. Phys..

[3] Jiao D., Ni L., Zhu X., Zhe J., Zhao Z., Lyu Y., Liu Z. (2019). Measuring gaps using planar inductive sensors based on calculating mutual inductance. Sens. Actuators A Phys..

[4] Hikmat O.F., Ali M.S.M. (2017). RF MEMS Inductors and Their Applications—A Review. J. Microelectromechan. Syst..

[5] Casha O., Grech I., Micallef J., Gatt E., Morche D. (2011). RF VCO tuning using MEMS piezoelectric actuated variable inductors. Analog Integr. Circuits Signal Process..

[6] Yokoyama Y., Fukushige T., Hata S., Masu K., Shimokohbe A. (2003). On-chip variable inductor using microelectromechanical systems technology. Jpn. J. Appl. Phys..

[7] Chang S., Sivoththaman S. (2006). A Tunable RF MEMS Inductor on Silicon Incorporating an Amorphous Silicon Bimorph in a Low-Temperature Process. IEEE Electron. Device Lett..

[8] Zine-El-Abidine I., Okoniewski M., McRory J.G. (2005). Tunable radio frequency MEMS inductors with thermal bimorph actuators. J. Micromechanics Microeng..

[9] Zhang J., Chen D., Filippov D.A., Geng S., Li K., Zhang Q., Jiang L., Wang X., Zhu W., Cao L. (2019). Theory of tunable magnetoelectric inductors in ferrite-piezoelectric layered composite. J. Phys. D Appl. Phys..

[10] Chen H., Wang X., Gao Y., Shi X., Wang Z., Sun N., Zaeimbashi M., Liang X., He Y., Dong C. (2020). Integrated Tunable Magnetoelectric RF Inductors. IEEE Trans. Microw. Theory Tech..

[11] Wang Z., Wen T., Su W., Hu C., Chen Y., Hu Z., Wu J., Zhou Z., Liu M. (2020). Magnetic Sensor Based on Giant Magneto-Impedance in Commercial Inductors. IEEE Trans. Ind. Electron..

[12] Ning N., Li X., Fan J., Ng W., Xu Y., Qian X., Seet H. (2006). A Tunable Magnetic Inductor. IEEE Trans. Magn..

[13] Kim J.-I., Peroulis D. (2009). Tunable MEMS Spiral Inductors with Optimized RF Performance and Integrated Large-Displacement Electrothermal Actuators. IEEE Trans. Microw. Theory Tech..

[14] Zhou S., Sun X.-Q., Carr W. (1997). A micro variable inductor chip using MEMS relays. Proceedings of the International Conference on Solid State Sensors and Actuators (TRANSDUCERS).

[15] Zhou S., Sun X.-Q., Carr W.N. (1999). A monolithic variable inductor network using microrelays with combined thermal and electrostatic actuation. J. Micromechanics Microeng..

[16] Shirane A., Ito H., Ishihara N., Masu K. (2012). Planar Solenoidal Inductor in Radio Frequency Micro-Electro-Mechanical Systems Technology for Variable Inductor with Wide Tunable Range and High Quality Factor. Jpn. J. Appl. Phys..

[17] Gmati I.E., Calmon P.F., Boukabache A., Pons P., Fulcrand R., Pinon S., Boussetta H., Kallala M.A., Besbes K. (2011). Fabrication and evaluation of an on-chip liquid micro-variable inductor. J. Micromechanics Microeng..

[18] Sugawara H., Yokoyama Y., Gomi S., Ito H., Okada K., Hoshino H., Onodera H., Masu K. (2004). Variable RF inductor on Si CMOS chip. Jpn. J. Appl. Phys..

[19] Sugawara H., Yoshihara Y., Ito H., Okada K., Masu K. (2004). Wide-range RF variable inductor on Si CMOS chip with MEMS actuator. Proceedings of the 34th European Microwave Conference (EuMC).

[20] Okada K., Sugawara H., Ito H., Itoi K., Sato M., Abe H., Ito T., Masu K. (2006). On-Chip High-Q Variable Inductor Using Wafer-Level Chip-Scale Package Technology. IEEE Trans. Electron Devices.

[21] Lubecke V., Barber B., Chan E., Lopez D., Gross M., Gammel P. (2001). Self-Assembling MEMS Variable and Fixed RF Inductors. IEEE Trans. Microw. Theory Tech..

[22] Singh R., Singh E., Nalwa H. (2017). Inkjet printed nanomaterial based flexible radio frequency identification (RFID) tag sensors for the internet of nano things. RSC Adv..

[23] Huang R., Urban A., Jiao D., Zhe J., Choi J.-W. (2022). Inductive proximity sensors within a ceramic package manufactured by material extrusion of binder-coated zirconia. Sens. Actuators A Phys..

[24] Hamia R., Cordier C., Dolabdjian C. (2014). Eddy-current non-destructive testing system for the determination of crack orientation. NDT E Int..

[25] Yin Y., Liu Z., Zheng J., Chen L., Wu S., Wang S., Yan Z., Pan X. (2019). The Effects of Position on the Wear Debris Detection with Planar Inductor. Sensors.

[26] Mukhopadhyay S., Yamada S., Iwahara M. (2002). Inspection of electroplated materials—Performance comparison with planar meander and mesh type magnetic sensor. Int. J. Appl. Electromagn. Mech..

[27] Islam A., Islam S., Tulip F. (2013). Design and Optimization of Printed Circuit Board Inductors for Wireless Power Transfer System. Circuits Syst..

[28] Wu L., Xu S., Zhong Z., Mou C., Wang X. (2020). An Inductive Sensor for Two-Dimensional Displacement Measurement. Sensors.

[29] Moreton G., Meydan T., Williams P. (2018). Using finite element modeling and experimental methods to investigate planar coil sensor topologies for inductive measurement of displacement. AIP Adv..

[30] Aldoumani M., Yuce B., Zhu D. (2021). Using the Variable Geometry in a Planar Inductor for an Optimised Performance. Electronics.

[31] Fang D.M., Li X.H., Yuan Q., Zhang H.X. (2010). Design, Simulation, and Characterization of Variable Inductor With Electrostatic Actuation Fabricated by Using Surface Micromachining Technology. IEEE Trans. Electron. Devices.

[32] Kaziz S., Maamer B., Delhaye T., Tounsi F., Francis L.A., Flandre D. (2020). Tuning Range Comparison between Different Planar Inductors Layouts on PCB. Proceedings of the IEEE International Conference on Design & Test of Integrated Micro & Nano-Systems (DTS).

[33] Craninckx J., Steyaert M.S.J. (1997). A 1.8-GHz low-phase-noise CMOSVCO using optimized hollow spiral inductors. IEEE J. Solid-State Circuits.

[34] Lopez-Villegas J., Samitier J., Cane C., Losantos P., Bausells J. (2000). Improvement of the quality factor of RF integrated inductors by layout optimization. IEEE Trans. Microw. Theory Tech..

[35] Lazarus N., Meyer C.D., Bedair S.S. (2014). Fractal Inductors. IEEE Trans. Magn..

[36] Salvia J., Bain J.A., Yue C.P. (2020). Tunable on-chip inductors up to 5 GHz using patterned permalloy laminations. Proceedings of the IEEE International Electron Devices Meeting (IEDM).

[37] Sasak Y., Kotani K. (2012). Postfabrication Independent Inductance and Quality Factor Adjustments of On-Chip Inductors by Above-CMOS Processing for Rapid Prototyping of Radio Frequency System on Chips. Jpn. J. Appl. Phys..

[38] González J.L., Aragonés X., Molina M., Martineau B., Belot D. (2020). A comparison between grounded and floating shield inductors for mmW VCOs. Proceedings of the IEEE European Conference on Solid-State Circuits (ESSCIRC).

[39] Camley R.E., Celinski Z., Stamps R.L. (2016). Magnetism of Surfaces, Interfaces, and Nanoscale Materials. Handbook of Surface Science.

